# Knowledge of cervical cancer risk factors among Palestinian women: a national cross-sectional study

**DOI:** 10.1186/s12905-021-01510-2

**Published:** 2021-11-02

**Authors:** Mohamedraed Elshami, Mariam Thalji, Hanan Abukmail, Ibrahim Al-Slaibi, Mohammed Alser, Afnan Radaydeh, Alaa Alfuqaha, Salma Khader, Lana Khatib, Nour Fannoun, Bisan Ahmad, Lina Kassab, Hiba Khrishi, Deniz Elhussaini, Nour Abed, Aya Nammari, Tumodir Abdallah, Zaina Alqudwa, Shahd Idais, Ghaid Tanbouz, Ma’alem Hajajreh, Hala Abu Selmiyh, Zakia Abo-Hajouj, Haya Hebi, Manar Zamel, Refqa Najeeb Skaik, Lama Hammoud, Saba Rjoub, Hadeel Ayesh, Toqa Rjoub, Rawan Zakout, Amany Alser, Nasser Abu-El-Noor, Bettina Bottcher

**Affiliations:** 1 Harvard Medical School, 25 Shattuck Street, Boston, MA 02115 USA; 2 Ministry of Health, Gaza, Palestine; 3 Faculty of Medicine, Al-Quds University, Jerusalem, Palestine; 4 Faculty of Medicine, Islamic University of Gaza, Gaza, Palestine; 5 Almakassed Hospital, Jerusalem, Palestine; 6 Faculty of Graduate Studies, An-Najah National University, Nablus, Palestine; 7 Faculty of Medicine, An-Najah National University, Nablus, Palestine; 8 Faculty of Pharmacy, Alazhar University of Gaza, Gaza, Palestine; 9 Faculty of Dentistry and Dental Surgery, Al-Quds University, Jerusalem, Palestine; 10 Faculty of Medicine, Alazhar University of Gaza, Gaza, Palestine; 11 Alia Hospital, Hebron, Palestine; 12 Al-Shiffa Hospital, Gaza, Palestine; 13 Faculty of Nursing, Islamic University of Gaza, Gaza, Palestine

**Keywords:** Cervical cancer, Prevention, Early detection, Survival, Risk factor, Awareness, Knowledge, Early presentation, Palestine

## Abstract

**Background:**

High awareness of cervical cancer (CC) risk factors is important to decrease the morbidity and mortality associated with CC. This study aimed to assess the knowledge level of Palestinian women about CC risk factors and to determine the factors associated with good knowledge.

**Methods:**

This was a national cross-sectional study. Adult women from hospitals, primary healthcare centers, and public spaces of 11 governorates in Palestine were recruited using a stratified convenience sampling. A translated-into-Arabic version of the validated CC awareness measure (CeCAM) was used to assess the knowledge about the 11 CC risk factors. For each correctly identified risk factor, the participant was given one point. The total score was calculated and was categorized into three categories: poor knowledge (0–3), fair knowledge (4–7), and good knowledge (8–11).

**Results:**

A total of 7223 participants completed the Arabic CeCAM (response rate = 89.3%) and 7058 questionnaires were included in the final analysis: 2655 from the Gaza Strip and 4403 from the West Bank and Jerusalem. Participants recruited from the Gaza Strip were younger, getting lower monthly incomes, and with less chronic diseases than participants recruited from the West Bank and Jerusalem. The most frequently identified risk factor was ‘having a weakened immune system’ (n = 5458, 77.3%) followed by ‘infection with a sexually transmitted infection’ (n = 5388, 76.3%). The least identified risk factor was ‘having many children’ (n = 1597, 22.6%). Only 1670 women (23.7%) had good knowledge of CC risk factors. Women living in the Gaza Strip were more likely than women living in the West Bank and Jerusalem to have good knowledge (25.2% vs 22.7%). Completing a secondary or diploma degree, being employed, and having a monthly income of ≥ 1450 NIS (around $450) were all associated with lower likelihood of having good knowledge of CC risk factors. Conversely, knowing someone with cancer was associated with higher likelihood of having good knowledge.

**Conclusion:**

The overall awareness of CC risk factors was low. There is a substantial need to establish educational programs to promote Palestinian women’s awareness of CC.

**Supplementary Information:**

The online version contains supplementary material available at 10.1186/s12905-021-01510-2.

## Introduction

Cervical cancer (CC) is the most commonly diagnosed gynecological cancer and one of the leading causes of cancer-related deaths in women worldwide [1, 2]. Globally, over 600,000 new cases and 300,000 deaths were estimated for CC in 2020 [1]. Half of these deaths occurred in countries of low and medium human development indices [1]. In Palestine, a lower-middle-income country, CC is the third most common gynecological cancer with an age-standardized incidence rate of 2.5 per 100,000 females [3–5]. CC in Palestine has a higher age-standardized mortality rate than other countries in the region [5]. This could be linked to the lack of an efficient screening program and diagnosis at later stages.

One of the main factors contributing to mortality of CC is diagnosis at a late stage [6, 7]. This could be a result of several factors including low awareness of CC symptoms and risk factors as well as limited access to healthcare facilities particularly in low- and middle-income countries [8–12]. There are many key factors that can increase the risk of CC development. The most significant risk factor of CC is infection with human papillomavirus (HPV) [13–16]. HPV type 16 and 18 are high-risk sexually transmitted viruses and are responsible for more than 70% of CC cases [14–16]. Other behavioral and sexual factors that also may contribute to CC development include multiple sexual partners, early age of sexual intercourse, multiparty, sexual intercourse with an uncircumcised man, smoking and poor personal hygiene [17–22].

In Palestine, where there is no national screening program for CC, raising public awareness is crucial to reduce morbidity and mortality of CC. Good awareness of CC risk factors plays an essential role in early detection and thus improved prognosis [6, 9]. Women who have good knowledge of CC risk factors are better able to recognize themselves as high-risk candidates to get the disease and therefore, they might seek medical advice earlier. Furthermore, women, who are aware to be at high-risk, are better equipped to adopt behaviors to reduce their probability of developing CC [23–25].

This national study aimed to: (1) assess Palestinian women’s level of knowledge of CC risk factors, (2) identify the factors associated with a good knowledge level, and (3) compare the knowledge among women from the Gaza Strip vs. the West Bank and Jerusalem (WBJ).

## Materials and methods

### Study design, setting and population

A national cross-sectional study was conducted between July 2019 and March 2020 in Palestine. The Palestinian Ministry of Health (MoH) hospitals and primary healthcare centers (PHCs) are the main entry sites for healthcare services in Palestine. These are distributed in two main geographical areas: (1) the Gaza Strip and (2) the WBJ. Therefore, governmental general hospitals with a bed capacity of more than 100 and PHCs with level four services (i.e., providing all primary healthcare services) were targeted to recruit participants into the study. Additionally, public spaces in the same governorates of hospitals and PHCs were involved, including markets, downtowns, mosques, churches, parks, malls, and restaurants.

In 2019, the estimated female population in Palestine was 2.45 million with about half of them in the reproductive age between 15 to 49 years [26]. Therefore, adult women aged 18 years or older were the target population and were invited to participate in the study. Potential participants were excluded if they had a citizenship other than Palestinian, were visiting the oncology departments, or were working or studying in a health-related field.

### Sampling methods

The data collection process took place in 11 hospitals, 12 PHCs as well as 11 public spaces across Palestine. The hospitals had bed capacities of over 100, while the PHCs offered all services to the general Palestinian public. These sites were located across Palestine in different governorates covering a wide geographical area and were chosen for recruitment of participants by stratified convenience sampling.

### Questionnaire and data collection

A translated-into-Arabic version of the validated Cervical Cancer Awareness Measure (CeCAM) was used [9]. The questionnaire consisted of two sections. The first section included socio-demographic questions. The second section comprised 11 questions based on a 5-point Likert scale (1 = strongly disagree, 5 = strongly agree) to assess the knowledge of CC risk factors.

The translation and adaptation of the questionnaire were performed based on World Health Organization recommendations [27]. The questionnaire was translated from English to Arabic by two healthcare professionals fluent in both languages and then back-translated into English by another two healthcare professionals who were also fluent in both languages. All healthcare professionals involved in this process had relevant clinical and research experience in gynecology, public health, and survey design.

A few items were adapted from the original CeCAM and were modified in the Arabic version to make them more culturally accepted in Palestine. ‘Having a sexual partner who is not circumcised’ was modified into ‘having a husband who is not circumcised’. Similarly, ‘having a sexual partner with many previous partners’ was modified into ‘having a husband with many previous partners’. In addition, ‘starting to have sex at a young age (before age 17)’ was modified into ‘being married at a young age (before age 17)’.

The Arabic CeCAM was modified for the purposes of this study. To minimize the possibility of participants answering questions at random, the original questions with yes/no/unknown responses were modified into 5-point Likert scale questions. Meanwhile, the participants’ responses were then converted to correct/incorrect responses similar to what was done in previous studies [28–31].

A pilot study was conducted with 130 respondents to test the clarity of the items of the Arabic CeCAM version. These responses were not included in the final analysis. The Cronbach’s Alpha showed that the questionnaire had an acceptable internal consistency (α = 0.72).

Well-trained data collectors with a medical background conducted face-to-face interviews with the recruited participants for completion of the Arabic CeCAM. Data were collected utilizing the secure, user-friendly data collection tool ‘Kobo Toolbox’ that is accessed via smartphones [32].

### Statistical analysis

Participant characteristics were summarized utilizing descriptive statistics. Continuous non-normally distributed variables were described using the median and interquartile range. Frequencies and percentages were utilized to summarize categorical variables. To reflect the age-associated risk of CC, age was categorized into three groups: 18–20 years, 21–40 years (at-risk group), and ≥ 41 years [9]. A monthly income of 1450 NIS (about $450) was the minimum wage in Palestine at the time of data collection [33]. Therefore, participants were categorized into two categories: ≥ 1450 NIS and < 1450 NIS. Baseline characteristics of participants from the WBJ vs. the Gaza Strip were compared using Pearson's Chi-square test if they were categorical or Kruskal–Wallis test if they were continuous.

For questions asking about CC risk factors, answering with ‘strongly agree’ and ‘agree’ was considered as a correct answer, whereas answering with ‘strongly disagree’, ‘disagree’, or ‘not sure’ was considered as an incorrect answer. Recognizing each CC risk factor was described using frequencies and percentages with comparisons utilizing Pearson's Chi-square test. This was followed by bivariable and multivariable logistic regression analyses. The model of the multivariable analysis adjusted for factors of socioeconomic status including age, educational level, occupation, monthly income, residency, and marital status. In addition, the model adjusted for other factors including having a chronic disease, knowing someone with cancer, and site of data collection. The model was pre-specified based on previous studies [9, 34–36]. Results of all bivariable logistic regression analyses were provided in Additional file 1.

To evaluate the knowledge level of CC risk factors, a scoring system was used. Similar scoring systems had been adopted in previous studies [24, 28]. For each correctly identified risk factor, the participant was given one point. The total score was then calculated (ranging from 0 to 11) and was categorized into three categories: poor knowledge (0–3), fair knowledge (4–7), and good knowledge (8–11). The knowledge level between the participants from the Gaza Strip and the WBJ was compared using Pearson's Chi-square test. Bivariable and multivariable logistic regression analyses were used to test the association between participants’ characteristics and having a good knowledge level.

Complete case analysis was used to handle missing data (i.e., cases with incomplete data were excluded from the analysis; a total of 135 cases). The missing data were completely random and unrelated to the study variables. Data were analyzed using Stata software version 16.0 (StataCorp, College Station, Texas, United States).

## Results

### Participant characteristics

A total of 7223 participants, out of 8086 approached, completed the questionnaire (response rate = 89.3%). The final analysis included 7058 questionnaires (30 did not meet inclusion criteria and 135 had missing values); 4403 from the WBJ and 2655 from the Gaza Strip.

The median age [interquartile range] for all participants was 32.0 years [24.0, 42.0] (Table 1). Participants recruited from the Gaza Strip were younger, getting lower monthly income, and with less chronic diseases than participants recruited from the WBJ.

**Table 1:** Characteristics of study participants

Characteristic	Total (n = 7058)	Gaza strip (n = 2655)	WBJ (n = 4403)
Age, median [IQR]	32 [24, 42]	30 [24, 39]	33 [24, 44]
Age group, n (%)			
18–20	756 (10.7)	249 (9.4)	507 (11.5)
21–40	4331 (61.4)	1809 (68.1)	2522 (57.3)
41 or older	1971 (27.9)	597 (22.5)	1374 (31.2)
Educational level, n (%)			
Illiterate	127 (1.8)	37 (1.4)	90 (2.0)
Primary	409 (5.8)	127 (4.8)	282 (6.4)
Preparatory	1064 (15.1)	378 (14.2)	686 (15.6)
Secondary	2293 (32.5)	955 (36.0)	1338 (30.4)
Diploma	766 (10.9)	303 (11.4)	463 (10.5)
Bachelor	2261 (32.0)	817 (30.8)	1444 (32.8)
Postgraduate	138 (1.9)	38 (1.4)	100 (2.3)
Occupation, n (%)			
Housewife	4647 (65.8)	2008 (75.6)	2639 (59.9)
Employed	1476 (20.9)	348 (13.1)	1128 (25.6)
Retired	69 (1.0)	11 (0.4)	58 (1.3)
Student	866 (12.3)	288 (10.9)	578 (13.2)
Monthly income ≥ 1450 NIS, n (%)	4666 (66.1)	693 (26.1)	3973 (90.2)
Having a chronic disease, n (%)	1397 (19.8)	417 (15.7)	980 (22.3)
Knowing someone with cancer, n (%)	4083 (57.9)	1483 (55.9)	2600 (59.1)
Marital status, n (%)			
Single	1657 (23.4)	527 (19.8)	1130 (25.6)
Married	5058 (71.7)	2025 (76.3)	3033 (68.9)
Divorced	154 (2.2)	45 (1.7)	109 (2.5)
Widowed	189 (2.7)	58 (2.2)	131 (3.0)
Site of data collection			
Public spaces, n (%)	2695 (38.2)	863 (32.5)	1832 (41.7)
Hospitals, n (%)	1890 (26.8)	642 (24.2)	1248 (28.3)
Primary healthcare centers, n (%)	2473 (35.0)	1150 (43.3)	1323 (30.0)

n, number of participants; IQR, interquartile range; WBJ, West Bank and Jerusalem

### Good knowledge and its associated factors

Only 1670 women (23.7%) had a good knowledge of CC risk factors (Table 2). Women living in the Gaza Strip were more likely than women living in the WBJ to have good knowledge (25.2% vs 22.7%).

**Table 2:** Knowledge level among study participants

Level	Total n (%)	Gaza strip n (%)	WBJ n (%)	*p* value
Poor	1140 (16.1)	374 (14.1)	766 (17.4)	< 0.001
Fair	4248 (60.2)	1611 (60.7)	2637 (59.9)	
Good	1670 (23.7)	670 (25.2)	1000 (22.7)	

n, number of participants; WBJ, West Bank and Jerusalem

On the multivariable analysis, completing secondary or diploma degree, being employed, and having a monthly income of ≥ 1450 NIS were all associated with a decrease in the odds of having good knowledge of CC risk factors (Table 3). On the other hand, knowing someone with cancer was associated with an increase in the odds of having good knowledge.

**Table 3:** Association between having a good knowledge and sociodemographic factors

Characteristic	Good knowledge				
	n (%)	COR (95% CI)	*p* value	AOR (95% CI)^a^	*p* value
*Age group*					
18–20	157 (9.4)	Ref	Ref	Ref	Ref
21–40	1016 (60.8)	1.17 (0.97–1.41)	0.11	1.17 (0.92–1.48)	0.20
41 or older	497 (29.8)	1.29 (1.05–1.58)	0.015	129 (0.98–1.69)	0.07
*Educational level*					
Illiterate	40 (2.4)	Ref	Ref	Ref	Ref
Primary	112 (6.7)	0.82 (0.53–1.26)	0.37	0.75 (0.48–1.17)	0.21
Preparatory	260 (15.6)	0.70 (0.47–1.05)	0.08	0.67 (0.44–1.00)	0.051
Secondary	513 (30.7)	0.63 (0.43–0.92)	0.018	0.63 (0.42–0.94)	0.023
Diploma	162 (9.7)	0.58 (0.39–0.88)	0.010	0.63 (0.41–0.97)	0.035
Bachelor	540 (32.3)	0.68 (0.46–1.00)	0.053	0.76 (0.50–1.14)	0.19
Postgraduate	43 (2.6)	0.98 (0.59–1.66)	0.95	1.17 (0.67–2.04)	0.57
*Occupation*					
Housewife	1144 (68.5)	Ref	Ref	Ref	Ref
Employed	316 (18.9)	0.83 (0.72–0.96)	0.012	0.81 (0.68–0.96)	0.016
Retired	13 (0.8)	0.71 (0.39–1.30)	0.27	0.76 (0.40–1.43)	0.39
Student	197 (11.8)	0.90 (0.76–1.07)	0.24	1.05 (0.82–1.35)	0.71
*Monthly income*					
< 1450 NIS	619 (37.1)	Ref	Ref	Ref	Ref
≥ 1450 NIS	1051 (62.9)	0.83 (0.74–0.93)	0.002	0.85 (0.72–0.99)	0.038
*Marital status*					
Single	362 (21.7)	Ref	Ref	Ref	Ref
Married	1226 (73.4)	1.14 (1.00–1.31)	0.047	1.06 (0.89–1.26)	0.55
Divorced	39 (2.3)	1.21 (0.83–1.78)	0.32	1.17 (0.79–1.74)	0.44
Widowed	43 (2.6)	1.05 (0.74–1.51)	0.78	0.85 (0.57–1.26)	0.41
*Residency*					
Gaza strip	670 (40.1)	Ref	Ref	Ref	Ref
WBJ	1000 (59.9)	0.87 (0.78–0.97)	0.016	0.97 (0.83–1.13)	0.66
*Having a chronic disease*					
No	1314 (78.7)	Ref	Ref	Ref	Ref
Yes	356 (21.3)	1.13 (0.99––1.30)	0.07	1.05 (0.90–1.22)	0.58
*Knowing someone with cancer*					
No	611 (36.6)	Ref	Ref	Ref	Ref
Yes	1059 (63.4)	1.34 (1.20–1.51)	< 0.001	1.34 (1.19–1.50)	< 0.001
*Site of data collection*					
Public spaces	636 (38.1)	Ref	Ref	Ref	Ref
Hospitals	442 (26.5)	0.99 (0.86–1.14)	0.87	0.94 (0.81–1.10)	0.45
Primary healthcare centers	592 (35.4)	1.02 (0.90–1.16)	0.78	0.96 (0.84–1.10)	0.58

COR, crude odds ratio; AOR, adjusted odds ratio; CI, confidence interval; WBJ, West Bank and Jerusalem

^a^Adjusted for age-group, educational level, occupation, monthly income, marital status, residency, having a chronic disease, knowing someone with cancer, and site of data collection

### Recognition of CC risk factors in the Gaza strip versus the WBJ

Among all participants, the most frequently recognized risk factor was ‘having a weakened immune system’ (n = 5458, 77.3%) followed by ‘infection with a sexually transmitted infection (STI)’ (n = 5388, 76.3%) (Table 4). These risk factors were also the most recognized factors in both the Gaza Strip and WBJ. The least recognized risk factors were ‘having many children’ (n = 1597, 22.6%) and ‘being married at a young age’ (n = 2197, 31.1%).

**Table 4:** Recognition of cervical cancer risk factors

Risk factor	Total (n = 7058) n (%)	Gaza strip (n = 2655) n (%)	WBJ (n = 4403) n (%)	*p* value
Having a weakened immune system	5458 (77.3)	2139 (80.6)	3319 (75.4)	< 0.001
Infection with a sexually transmitted infection	5388 (76.3)	2132 (80.3)	3256 (73.9)	< 0.001
Infection with human papillomavirus (HPV)	4693 (66.5)	1977 (74.5)	2716 (61.7)	< 0.001
Having a relative with cervical cancer	4250 (60.2)	1538 (57.9)	2712 (61.6)	0.002
Long term use of the contraceptive pill	4236 (60.0)	1620 (61.0)	2616 (59.4)	0.18
Smoking any cigarettes at all	4167 (59.0)	1600 (60.3)	2567 (58.3)	0.10
Not going for regular smear (Pap) tests	3543 (50.2)	1507 (56.8)	2036 (46.2)	< 0.001
Having a husband who is not circumcised	2818 (39.9)	1127 (42.4)	1691 (38.4)	< 0.001
Having a husband with many previous partners	2562 (36.3)	842 (31.7)	1720 (39.1)	< 0.001
Being married at a young age (before age 17)	2197 (31.1)	779 (29.3)	1418 (32.2)	0.012
Having many children (five or more)	1597 (22.6)	559 (21.1)	1038 (23.6)	0.014

n, number of participants; WBJ, West Bank and Jerusalem

The Chi-square test showed that participants from the Gaza Strip had a higher likelihood than participants from the WBJ to recognize ‘having a weakened immune system’, ‘infection with a sexually transmitted infection (STI)’, ‘infection with HPV’, ‘not going for regular Pap smears’, and ‘having uncircumcised husband’. On the other hand, participants from the WBJ were more likely to recognize ‘having a relative with CC’, ‘having a husband with many previous partners’, ‘being married at a young age’, and ‘having many children’ as risk factors for CC.

### Association between recognizing CC risk factors and socioeconomic status

On the multivariable analysis, women with age-related risk of CC (aged 21–40 years) were less likely than younger women (aged 18–20 years) to recognize ‘infection with an STI’ (OR = 0.71, 95% CI 0.56–0.91), ‘infection with HPV’ (OR = 0.76, 95% CI 0.61–0.96), and ‘not going to regular Pap smears’ (OR = 0.75, 95% CI 0.61–0.91) as risk factors for CC (Tables 5, 6).

**Table 5:** Multivariable logistic regression analyzing the association between the recognition of the most identified risk factors and sociodemographic factors

Characteristic	Having a weakened immune system (n = 5458)			Infection with a sexually transmitted infection (n = 5388)			Infection with HPV (n = 4693)		
	n (%)	AOR (95% CI)^a^	*p* value	n (%)	AOR (95% CI)^a^	*p* value	n (%)	AOR (95% CI)^a^	*p* value
*Age group*									
18–20	560 (10.3)	Ref	Ref	607 (11.3)	Ref	Ref	574 (12.2)	Ref	Ref
21–40	3355 (61.5)	0.97 (0.77–1.22)	0.81	3329 (61.8)	0.71 (0.56–0.91)	0.006	2878 (61.3)	0.76 (0.61–0.96)	0.018
41 or older	1543 (28.3)	1.16 (0.89–1.51)	0.29	1452 (26.9)	0.74 (0.56–0.97)	0.029	1241 (26.4)	0.81 (0.63–1.04)	0.10
*Educational level*									
Illiterate	78 (1.4)	Ref	Ref	80 (1.5)	Ref	Ref	73 (1.6)	Ref	Ref
Primary	294 (5.4)	1.42 (0.93–2.19)	0.11	265 (4.9)	1.00 (0.66–1.53)	0.99	241 (5.1)	1.08 (0.71–1.63)	0.73
Preparatory	828 (15.2)	2.01 (1.34–3.00)	0.001	801 (14.9)	1.62 (1.09–2.41)	0.018	664 (14.1)	1.22 (0.83–1.80)	0.31
Secondary	1805 (33.1)	2.44 (1.64–3.62)	< 0.001	1787 (33.2)	1.90 (1.28–2.81)	0.001	1513 (32.2)	1.29 (0.88–1.89)	0.18
Diploma	559 (10.2)	2.08 (1.37–3.17)	0.001	563 (10.4)	1.84 (1.21–2.81)	0.004	511 (10.9)	1.52 (1.01–2.28)	0.043
Bachelor	1777 (32.6)	2.92 (1.94–4.39)	< 0.001	1778 (33.0)	2.40 (1.60–3.60)	< 0.001	1589 (33.9)	1.69 (1.14–2.50)	0.009
Postgraduate	117 (2.1)	4.63 (2.48–8.65)	< 0.001	114 (2.1)	3.64 (1.99–6.66)	< 0.001	102 (2.2)	2.47 (1.42–4.28)	0.001
*Occupation*									
Housewife	3653 (66.9)	Ref	Ref	3591 (66.6)	Ref	Ref	3043 (64.8)	Ref	Ref
Employed	1122 (20.6)	0.92 (0.78–1.09)	0.35	1083 (20.1)	0.72 (0.61–0.85)	< 0.001	945 (20.1)	0.80 (0.69–0.93)	0.004
Retired	39 (0.7)	0.38 (0.22–0.65)	< 0.001	32 (0.6)	0.28 (0.17–0.47)	< 0.001	34 (0.7)	0.53 (0.32–0.88)	0.014
Student	644 (11.8)	1.09 (0.85–1.40)	0.49	682 (12.7)	0.89 (0.69–1.15)	0.38	671 (14.3)	1.31 (1.03–1.67)	0.027
*Monthly income*									
< 1450 NIS	1867 (34.2)	Ref	Ref	1851 (34.4)	Ref	Ref	1700 (36.2)	Ref	Ref
≥ 1450 NIS	3591 (65.8)	1.22 (1.03–1.44)	0.020	3537 (65.6)	1.19 (1.01–1.40)	0.041	2993 (63.8)	0.97 (0.84–1.13)	0.74
*Residency*									
Gaza Strip	2139 (39.2)	Ref	Ref	2132 (39.6)	Ref	Ref	1977 (42.1)	Ref	Ref
WBJ	3319 (60.8)	0.70 (0.59–0.82)	< 0.001	3256 (60.4)	0.67 (0.57–0.79)	< 0.001	2716 (57.9)	0.53 (0.46–0.61)	< 0.001
*Having a chronic disease*									
No	4358 (79.8)	Ref	Ref	4344 (80.6)	Ref	Ref	3815 (81.3)	Ref	Ref
Yes	1100 (20.2)	1.17 (0.99–1.38)	0.07	1044 (19.4)	1.12 (0.96–1.31)	0.15	878 (18.7)	0.99 (0.86–1.14)	0.88
*Knowing someone with cancer*									
No	2180 (39.9)	Ref	Ref	2206 (40.9)	Ref	Ref	1891 (40.3)	Ref	Ref
Yes	3278 (60.1)	1.41 (1.25–1.58)	< 0.001	3182 (59.1)	1.15 (1.03–1.29)	0.015	2802 (59.7)	1.23 (1.11–1.37)	< 0.001
*Marital status*									
Single	1183 (21.7)	Ref	Ref	1229 (22.8)	Ref	Ref	1168 (24.9)	Ref	Ref
Married	4021 (73.7)	1.55 (1.30–1.83)	< 0.001	3918 (72.7)	1.38 (1.17–1.64)	< 0.001	3306 (70.4)	1.08 (0.92–1.26)	0.36
Divorced	112 (2.1)	1.18 (0.80–1.74)	0.40	114 (2.1)	1.25 (0.84–1.85)	0.27	102 (2.2)	1.11 (0.77–1.60)	0.59
Widowed	142 (2.6)	1.51 (1.02–2.23)	0.039	127 (2.4)	1.05 (0.73–1.51)	0.79	117 (2.5)	1.06 (0.75–1.50)	0.75
*Site of data collection*									
Public spaces	2015 (36.9)	Ref	Ref	2113 (39.2)	Ref	Ref	1966 (41.9)	Ref	Ref
Hospitals	1423 (26.1)	1.04 (0.89–1.20)	0.64	1362 (25.3)	0.72 (0.62–0.84)	< 0.001	1216 (25.9)	0.73 (0.64–0.84)	< 0.001
Primary healthcare centers	2020 (37.0)	1.41 (1.22–1.63)	< 0.001	1913 (35.5)	0.86 (0.75–0.99)	0.040	1511 (32.2)	0.56 (0.49–0.64)	< 0.001

Characteristic	Having a relative with cervical cancer (n = 4250)			Long term use of the contraceptive pill (n = 4236)			Smoking any cigarettes at all (n = 4167)		
	n (%)	AOR (95% CI)^a^	*p* value	n (%)	AOR (95% CI)^a^	*p* value	n (%)	AOR (95% CI)^a^	*p* value
*Age group*									
18–20	418 (9.8)	Ref	Ref	446 (10.5)	Ref	Ref	405 (9.7)	Ref	Ref
21–40	2629 (61.9)	1.05 (0.86–1.29)	0.61	2588 (61.1)	0.96 (0.79–1.17)	0.71	2566 (61.6)	1.02 (0.83–1.24)	0.88
41 or older	1203 (28.3)	1.01 (0.81–1.28)	0.90	1202 (28.4)	1.02 (0.81–1.28)	0.90	1196 (28.7)	0.96 (0.76–1.21)	0.72
*Educational level*									
Illiterate	78 (1.8)	Ref	Ref	77 (1.8)	Ref	Ref	82 (2.0)	Ref	Ref
Primary	264 (6.2)	1.02 (0.67–1.55)	0.93	240 (5.7)	0.89 (0.59–1.34)	0.56	258 (6.2)	0.89 (0.59–1.36)	0.61
Preparatory	685 (16.1)	0.96 (0.65–1.42)	0.85	631 (14.9)	0.91 (0.62–1.34)	0.63	701 (16.8)	0.99 (0.67–1.48)	0.99
Secondary	1387 (32.6)	0.86 (0.58–1.25)	0.42	1346 (31.8)	0.92 (0.63–1.35)	0.67	1371 (32.9)	0.83 (0.56–1.22)	0.34
Diploma	414 (9.7)	0.72 (0.48–1.08)	0.12	428 (10.1)	0.87 (0.58–1.29)	0.49	429 (10.3)	0.79 (0.53–1.19)	0.26
Bachelor	1338 (31.5)	0.88 (0.59–1.30)	0.52	1417 (33.5)	1.13 (0.76–1.66)	0.55	1245 (29.9)	0.78 (0.52–1.16)	0.22
Postgraduate	84 (2.0)	0.91 (0.54–1.55)	0.74	97 (2.3)	1.56 (0.91–2.66)	0.11	81 (1.9)	0.94 (0.55–1.58)	0.81
*Occupation*									
Housewife	2900 (68.2)	Ref	Ref	2776 (65.5)	Ref	Ref	2884 (69.2)	Ref	Ref
Employed	854 (20.1)	0.91 (0.79–1.05)	0.21	907 (21.4)	1.01 (0.88–1.17)	0.85	796 (19.1)	0.93 (0.80–1.07)	0.29
Retired	29 (0.7)	0.63 (0.38–1.06)	0.08	33 (0.8)	0.66 (0.40–1.10)	0.11	33 (0.8)	0.79 (0.47–1.31)	0.35
Student	467 (11.0)	0.88 (0.71–1.09)	0.23	520 (12.3)	1.08 (0.87–1.34)	0.47	454 (10.9)	1.08 (0.88–1.34)	0.46
*Monthly income*									
< 1450 NIS	1394 (32.8)	Ref	Ref	1413 (33.4)	Ref	Ref	1476 (35.4)	Ref	Ref
≥ 1450 NIS	2856 (67.2)	1.13 (0.98–1.30)	0.09	2823 (66.6)	1.13 (0.98–1.30)	0.09	2691 (64.6)	0.93 (0.81–1.08)	0.34
*Residency*									
Gaza Strip	1538 (36.2)	Ref	Ref	1620 (38.2)	Ref	Ref	1600 (38.4)	Ref	Ref
WBJ	2712 (63.8)	1.19 (1.04–1.36)	0.011	2616 (61.8)	0.85 (0.74–0.98)	0.021	2567 (61.6)	1.04 (0.91–1.19)	0.59
*Having a chronic disease*									
No	3408 (80.2)	Ref	Ref	3367 (79.5)	Ref	Ref	3314 (79.5)	Ref	Ref
Yes	842 (19.8)	0.91 (0.79–1.04)	0.18	869 (20.5)	1.14 (0.99–1.30)	0.07	853 (20.5)	0.98 (0.86–1.13)	0.81
*Knowing someone with cancer*									
No	1671 (39.3)	Ref	Ref	1666 (39.3)	Ref	Ref	1684 (40.4)	Ref	Ref
Yes	2579 (60.7)	1.33 (1.20–1.47)	< 0.001	2570 (60.7)	1.28 (1.16–1.42)	< 0.001	2483 (59.6)	1.21 (1.10–1.34)	< 0.001
*Marital status*									
Single	895 (21.1)	Ref	Ref	954 (22.5)	Ref	Ref	829 (19.9)	Ref	Ref
Married	3165 (74.5)	1.22 (1.05–1.42)	0.009	3073 (72.5)	1.26 (1.08–1.46)	0.003	3121 (74.9)	1.41 (1.21–1.63)	< 0.001
Divorced	80 (1.9)	0.88 (0.62–1.24)	0.45	96 (2.3)	1.33 (0.94–1.90)	0.11	90 (2.2)	1.37 (0.97–1.94)	0.07
Widowed	110 (2.6)	1.06 (0.76–1.49)	0.72	113 (2.7)	1.19 (0.85–1.67)	0.31	127 (3.0)	1.81 (1.28–2.57)	0.001
*Site of data collection*									
Public spaces	1531 (36.0)	Ref	Ref	1666 (39.3)	Ref	Ref	1432 (34.4)	Ref	Ref
Hospitals	1050 (24.7)	0.87 (0.77–0.99)	0.037	1091 (25.8)	0.85 (0.75–0.97)	0.016	1095 (26.3)	1.11 (0.98–1.26)	0.10
Primary healthcare centers	1669 (39.3)	1.52 (1.34–1.72)	< 0.001	1479 (34.9)	0.92 (0.82–1.04)	0.18	1640 (39.4)	1.58 (1.40–1.79)	< 0.001

n, number of participants; AOR, adjusted odds ratio; CI, confidence interval; WBJ, West Bank and Jerusalem; HPV, human papillomavirus

^a^Adjusted for age-group, educational level, occupation, monthly income, marital status, residency, having a chronic disease, knowing someone with cancer, and site of data collection

**Table 6:** Multivariable logistic regression analyizing the association between the recognition of other risk factors and sociodemographic factors

Characteristic	Not going for regular smear (pap) tests (n = 3543)			Having a husband who is not circumcised (n = 2818)			Having a husband with many previous partners (n = 2562)		
	n (%)	AOR (95% CI)^a^	*p* value	n (%)	AOR (95% CI)^a^	*p* value	n (%)	AOR (95% CI)^a^	*p* value
*Age group*									
18–20	407 (11.5)	Ref	Ref	346 (12.3)	Ref	Ref	266 (10.4)	Ref	Ref
21–40	2188 (61.8)	0.75 (0.61–0.91)	0.004	1752 (62.2)	0.88 (0.72–1.07)	0.19	1524 (59.5)	1.02 (0.83–1.25)	0.86
41 or older	948 (26.8)	0.72 (0.57–0.90)	0.005	720 (25.6)	0.79 (0.63–0.99)	0.043	772 (30.1)	1.04 (0.82–1.32)	0.73
*Educational level*									
Illiterate	64 (1.8)	Ref	Ref	55 (2.0)	Ref	Ref	64 (2.5)	Ref	Ref
Primary	201 (5.7)	0.87 (0.58–1.31)	0.50	157 (5.6)	0.84 (0.56–1.26)	0.40	192 (7.5)	0.85 (0.56–1.27)	0.42
Preparatory	569 (16.1)	0.98 (0.67–1.44)	0.94	409 (14.5)	0.80 (0.55–1.17)	0.26	433 (16.9)	0.69 (0.47–1.01)	0.06
Secondary	1137 (32.1)	0.82 (0.56–1.19)	0.29	913 (32.4)	0.79 (0.54–1.15)	0.23	801 (31.3)	0.57 (0.39–0.83)	0.003
Diploma	353 (10.0)	0.80 (0.54–1.18)	0.26	270 (9.6)	0.71 (0.47–1.05)	0.09	259 (10.1)	0.56 (0.38–0.83)	0.004
Bachelor	1153 (32.5)	1.00 (0.68–1.47)	0.99	956 (33.9)	0.90 (0.62–1.33)	0.61	759 (29.6)	0.56 (0.38–0.83)	0.003
Postgraduate	66 (1.9)	0.97 (0.58–1.63)	0.92	58 (2.1)	1.01 (0.60–1.68)	0.98	54 (2.1)	0.77 (0.46–1.28)	0.31
*Occupation*									
Unemployed/Housewife	2414 (68.1)	Ref	Ref	1843 (65.4)	Ref	Ref	1734 (67.7)	Ref	Ref
Employed	674 (19.0)	0.87 (0.75–0.99)	0.047	545 (19.3)	0.89 (0.77–1.03)	0.11	486 (19.0)	0.87 (0.75–1.01)	0.06
Retired	22 (0.6)	0.62 (0.36–1.06)	0.08	19 (0.7)	0.77 (0.44–1.34)	0.35	29 (1.1)	1.24 (0.74–2.06)	0.41
Student	433 (12.2)	0.85 (0.69–1.06)	0.15	411 (14.6)	1.32 (1.07–1.63)	0.011	313 (12.2)	1.19 (0.96–1.49)	0.12
*Monthly income*									
< 1450 NIS	1369 (38.6)	Ref	Ref	1007 (35.7)	Ref	Ref	834 (32.6)	Ref	Ref
≥ 1450 NIS	2174 (61.4)	0.80 (0.70–0.92)	0.002	1811 (64.3)	0.92 (0.80–1.05)	0.23	1728 (67.4)	0.84 (0.73–0.97)	0.021
*Residency*									
Gaza Strip	1507 (42.5)	Ref	Ref	1127 (40.0)	Ref	Ref	842 (32.9)	Ref	Ref
WBJ	2036 (57.5)	0.79 (0.69–0.90)	0.001	1691 (60.0)	0.89 (0.78–1.02)	0.09	1720 (67.1)	1.50 (1.30–1.72)	< 0.001
*Having a chronic disease*									
No	2871 (81.0)	Ref	Ref	2313 (82.1)	Ref	Ref	2011 (78.5)	Ref	Ref
Yes	672 (19.0)	0.91 (0.80–1.05)	0.19	505 (17.9)	0.88 (0.77–1.01)	0.07	551 (21.5)	1.01 (0.88–1.16)	0.87
*Knowing someone with cancer*									
No	1369 (38.6)	Ref	Ref	1150 (40.8)	Ref	Ref	1039 (40.6)	Ref	Ref
Yes	2174 (61.4)	1.40 (1.27–1.55)	< 0.001	1668 (59.2)	1.12 (1.02–1.24)	0.022	1523 (59.4)	1.09 (0.98–1.20)	0.12
*Marital status*									
Single	811 (22.9)	Ref	Ref	668 (23.7)	Ref	Ref	552 (21.5)	Ref	Ref
Married	2557 (72.2)	0.97 (0.84–1.13)	0.70	2007 (71.2)	1.23 (1.06–1.43)	0.007	1880 (73.4)	1.20 (1.03–1.41)	0.019
Divorced	82 (2.3)	1.24 (0.87–1.75)	0.23	65 (2.3)	1.37 (0.97–1.94)	0.07	58 (2.3)	1.22 (0.86–1.74)	0.27
Widowed	93 (2.6)	1.00 (0.71–1.40)	0.99	78 (2.8)	1.43 (1.02–2.01)	0.039	72 (2.8)	1.00 (0.71–1.42)	0.99
*Site of data collection*									
Public spaces	1266 (35.7)	Ref	Ref	1147 (40.7)	Ref	Ref	976 (38.1)	Ref	Ref
Hospitals	858 (24.2)	0.95 (0.84–1.08)	0.45	722 (25.6)	0.88 (0.78–1.01)	0.06	792 (30.9)	1.18 (1.03–1.34)	0.014
Primary healthcare centers	1419 (40.1)	1.47 (1.31–1.66)	< 0.001	949 (33.7)	0.85 (0.75–0.96)	0.007	794 (31.0)	0.80 (0.71–0.91)	0.001

Characteristic	Being married at a young age (before age 17) (n = 2197)			Having many children (five or more) (n = 1597)		
	n (%)	AOR (95% CI)^a^	*p* value	n (%)	AOR (95% CI)^a^	*p* value
*Age group*						
18–20	241 (11.0)	Ref	Ref	142 (8.9)	Ref	Ref
21–40	1318 (60.0)	1.03 (0.83–1.28)	0.77	970 (60.7)	1.21 (0.95–1.55)	0.12
41 or older	638 (29.0)	1.22 (0.96–1.56)	0.10	485 (30.4)	1.47 (1.11–1.94)	0.007
*Educational level*						
Illiterate	46 (2.1)	Ref	Ref	33 (2.1)	Ref	Ref
Primary	115 (5.2)	0.73 (0.48–1.12)	0.15	93 (5.8)	0.91 (0.57–1.45)	0.70
Preparatory	261 (11.9)	0.65 (0.44–0.97)	0.035	210 (13.1)	0.82 (0.53–1.28)	0.39
Secondary	646 (29.4)	0.82 (0.56–1.21)	0.32	470 (29.4)	0.93 (0.61–1.43)	0.76
Diploma	246 (11.2)	0.96 (0.63–1.45)	0.84	176 (11.0)	1.00 (0.64–1.58)	0.99
Bachelor	821 (37.4)	1.19 (0.80–1.77)	0.39	571 (35.8)	1.22 (0.79–1.89)	0.37
Postgraduate	62 (2.8)	1.67 (0.99–2.82)	0.054	44 (2.8)	1.59 (0.90–2.81)	0.11
*Occupation*						
Unemployed/housewife	1345 (61.2)	Ref	Ref	1007 (63.1)	Ref	Ref
Employed	517 (23.5)	0.99 (0.85–1.15)	0.86	389 (24.4)	1.04 (0.88–1.22)	0.66
Retired	32 (1.5)	1.39 (0.84–2.31)	0.20	22 (1.4)	1.12 (0.65–1.94)	0.67
Student	303 (13.8)	1.08 (0.86–1.35)	0.49	179 (11.2)	0.88 (0.68–1.13)	0.31
*Monthly income*						
< 1450 NIS	717 (32.6)	Ref	Ref	544 (34.1)	Ref	Ref
≥ 1450 NIS	1480 (67.4)	0.85 (0.73–0.99)	0.032	1053 (65.9)	0.77 (0.65–0.90)	0.002
*Residency*						
Gaza Strip	779 (35.5)	Ref	Ref	559 (35.0)	Ref	Ref
WBJ	1418 (64.5)	1.19 (1.03–1.37)	0.017	1038 (65.0)	1.30 (1.11–1.52)	0.001
*Having a chronic disease*						
No	1742 (79.3)	Ref	Ref	1268 (79.4)	Ref	Ref
Yes	455 (20.7)	1.11 (0.96–1.28)	0.15	329 (20.6)	1.00 (0.86–1.18)	0.96
*Knowing someone with cancer*						
No	920 (41.9)	Ref	Ref	710 (44.5)	Ref	Ref
Yes	1277 (58.1)	1.03 (0.93–1.15)	0.57	887 (55.5)	0.91 (0.81–1.02)	0.09
*Marital status*						
Single	579 (26.4)	Ref	Ref	396 (24.8)	Ref	Ref
Married	1497 (68.1)	0.92 (0.79–1.08)	0.30	1099 (68.8)	0.85 (0.72–1.01)	0.07
Divorced	58 (2.6)	1.17 (0.82–1.66)	0.39	51 (3.2)	1.36 (0.94–1.96)	0.10
Widowed	63 (2.9)	0.96 (0.67–1.36)	0.81	51 (3.2)	0.99 (0.68–1.45)	0.98
*Site of data collection*						
Public spaces	921 (41.9)	Ref	Ref	631 (39.5)	Ref	Ref
Hospitals	577 (26.3)	0.95 (0.83–1.09)	0.44	442 (27.7)	1.05 (0.91–1.22)	0.49
Primary healthcare centers	699 (31.8)	0.85 (0.75–0.97)	0.013	524 (32.8)	0.93 (0.81–1.07)	0.34

n, number of participants; AOR, adjusted odds ratio; CI, confidence interval; WBJ, West Bank and Jerusalem

^a^Adjusted for age-group, educational level, occupation, monthly income, marital status, residency, having a chronic disease, knowing someone with cancer, and site of data collection

Participants with a bachelor degree had a higher likelihood than illiterate participants to identify ‘having a weakened immune system’ (OR = 2.92, 95% CI 1.94–4.39), ‘infection with an STI’ (OR = 2.40, 95% CI 1.60–3.60), and ‘infection with HPV’ (OR = 1.69, 95% CI 1.14–2.50) as risk factors for CC. However, participants who had a bachelor degree were less likely to identify ‘having a husband with many previous partners’ (OR = 0.56, 95% CI 0.36–0.83) as a CC risk factor.

Married women were more likely than single women to recognize 7 out of 11 CC risk factors. Moreover, participants with a monthly income of ≥ 1450 NIS had a higher likelihood than participants with a lower monthly income to recognize ‘having a weakened immune system’ (OR = 1.22, 95% CI 1.03–1.44) and ‘infection with an STI’ (OR = 1.19, 95% CI 1.01–1.40) as risk factors for CC. Nonetheless, participants earning ≥ 1450 NIS had a lower likelihood to recognize other CC risk factors including ‘not going to regular Pap smears’, ‘having a husband with many previous partners’, ‘being married at a young age’, and ‘having many children’. In addition, employed women were less likely than unemployed or housewives to recognize ‘infection with an STI’ (OR = 0.72, 95% CI 0.61–0.85), ‘infection with HPV’ (OR = 0.80, 95% CI 0.69–0.93), and ‘not going to regular Pap smears’ (OR = 0.87, 95% CI 0.75–0.99).

### Association between recognizing CC risk factors and other participants’ characteristics

Women who knew someone with cancer were more likely than women who did not to identify all CC risk factors except ‘having a husband with many previous partners’, ‘being married at a young age’, and ‘having many children’ for which no differences were found.

Participants visiting hospitals were less likely than participants visiting public spaces to identify ‘infection with an STI’, ‘infection with HPV’, ‘having a relative with CC’, and ‘long term use of the contraceptive pill’. However, hospital visitors were more likely to identify ‘having a husband with many previous partners’ (OR = 1.18, 95% CI 1.03–1.34).

Participants visiting PHCs were less likely than participants visiting public spaces to identify ‘infection with an STI’, ‘infection with HPV’, ‘having a husband who is not circumcised’, ‘having a husband with many previous partners’, and ‘being married at a young age’ as risk factor for CC. However, visitors to PHCs were more likely to identify other CC risk factors including ‘having a weakened immune system’, ‘having a relative with CC’, ‘smoking any cigarettes at all’, and ‘not going for regular Pap smears’.

## Discussion

The overall awareness of CC risk factors in this study was low with only 23.7% of the participants having good knowledge. Knowing someone with cancer was associated with an increase in the odds of having good knowledge. Participants from the Gaza Strip demonstrated better knowledge than participants from the WBJ. 'Having a weakened immune system' was the most reported CC risk factor followed by ‘infection with an STI’. The least reported risk factors were ‘having many children’ and ‘being married at a young age’.

High awareness of CC risk factors could play an essential role in the prevention and early detection of CC [37, 38]. This study evaluated the Palestinian women’s level of knowledge of CC risk factors as a baseline for the implementation of future education programs. Such programs can be especially effective where no screening or prevention measures exist (e.g., HPV vaccine) as in Palestine.

### Knowledge level of CC risk factors and its associated factors

Good awareness of CC, early detection and treatment remain the cornerstones to improve CC survival outcomes especially in low- and middle-income countries [10, 12, 37, 39]. Only 23.7% of participants in this study had a good level of knowledge of CC risk factors, which is similar to reports from Tunisia, Libya, Qatar, and Oman [34, 35, 40, 41]. The relatively lower incidence and mortality rates in these Arab countries might have driven the health authorities to focus on educating women about other types of cancers that have higher rates (e.g., breast cancer) [42]. Education campaigns can be costly, and their funding is usually limited. However, the long-term investment in raising public awareness of CC risk factors may lead to prevention and early diagnosis of CC reducing the financial burden associated with treatment.

Low and colleagues reported better knowledge of CC risk factors among British women who knew someone with cancer, in concordance with this study and other studies in the United Kingdom [43, 44]. A possible explanation could be that women who know someone with cancer are expected to take care and accompany them during healthcare visits. Therefore, these women may come across more experience and knowledge about health-related topics. Furthermore, women’s concerns about someone’s health might lead them to read more about their diagnosis.

Married women were more likely than single women to recognize most of the CC risk factors in this study, which is in concordance with results of other studies [34, 35, 40]. Married women are expected to be more knowledgeable of topics related to reproductive and sexual health through their visits to healthcare facilities and, thus, also have higher chances to access more accurate information from healthcare professionals. In addition, married women may educate themselves by reading printed health materials distributed in clinics or by using internet resources. On the other hand, single women in conservative communities, such as Palestine, may feel inhibited to read or talk about sexual and reproductive health issues. In fact, the data collectors noticed this when they asked single women about risk factors related to sex, such as having a husband with many previous partners, marrying a husband who is not circumcised, and infection with an STI. Furthermore, although some health-related topics are part of the school curriculum in Palestine, topics around sexual health and CC are not included, potentially increasing the barriers of single women to address these topics. Therefore, education interventions should be tailored to address the emotional barriers of single women to promote their willingness to know more about reproductive health topics including CC.

Low socioeconomic status is one of the CC risk factors, raising the importance of improving the awareness of these factors in this group of women for prevention and early detection of CC [45]. In this study, being employed and having a high monthly income were associated with a decrease in the odds of having good knowledge. The decrease in the likelihood of having good knowledge associated with higher monthly income might only reflect the fact that women in the Gaza Strip know more than those in the WBJ but have less income. Furthermore, employed women were less likely to have a good knowledge, which might reflect that more single women might be employed than married women. In addition, those employed women might have less time to read about health-related topics and less involvement in social interactions where women talk about their own and their relatives’ experiences including those health issues. Another contributing factor might be that unemployment is higher in the Gaza Strip compared with the WBJ and this includes women, so that this might be another reflection of the generally better knowledge amongst women from Gaza compared with those from WBJ [46]. In contrast to this, previous studies showed that employed women and those having a high monthly income were more likely to have a good knowledge level of CC risk factors [35, 40, 47].

Higher education level was shown to be associated with more uptake of CC prevention and early detection strategies [48, 49]. Similar to previous studies on cancer awareness in the Gaza Strip, participants with only a secondary or diploma degree in this study showed lower likelihood to have good knowledge of CC risk factors [28–30]; highlighting the lack of such topics within the Palestinian school curricula. There is a need to revise school curricula to include a wider range of health-related topics. Kyle and colleagues demonstrated that a school-based educational intervention improved the recall and recognition of most of the cancer signs and symptoms even after six months from the intervention [50]. Raising such awareness among adolescents could be useful as this might shape their health-related behaviors in the future.

### Recognition of CC risk factors in the Gaza strip versus the WBJ

The participants from the Gaza Strip were more likely than participants from the WBJ to recognize 8 out of 11 CC risk factors. A possible contributing factor could be that living in extended families is more notable in the Gaza Strip. This could increase the likelihood of sharing and discussing health-related experiences or relatives’ stories, which may help in shaping women’s knowledge. Another form of interaction that could play a role in building women’s knowledge is the interaction with healthcare professionals. Women in the WBJ encounter several challenges in accessing healthcare facilities due to the Israeli checkpoints between geographical areas. These checkpoints restrict their movement and impede access to healthcare services [51, 52]. In contrast to this, movement within the Gaza Strip is easy and unrestricted for women, so that most women in the Gaza Strip can access healthcare facilities easily and shape their knowledge while communicating with healthcare providers [53, 54]. Moreover, women in the Gaza Strip have a relatively higher fertility rate among women of childbearing age (15–49 years) compared with those in the WBJ (4.5 vs 3.7 births per woman), therefore, they may be exposed to more experience in sexual and reproductive healthcare and associated health education [55].

### Recognizing CC risk factors

In this study, women recognized ‘infection with an STI’ more than ‘infection with HPV’. This is similar to findings among Libyan and British women [35, 43], which suggests that women are more aware of the link between CC and STIs than causative micro-organisms (e.g., HPV) as reported in the literature [35, 43, 56]. Future educational campaigns should highlight the role of HPV in CC etiology.

Having five or more children was the least risk factor reported in this study. This is similar to findings of other studies conducted in Libya, United States, India, Oman, and Malaysia [35, 47, 57–59]. A possible explanation for this could be that women’s thoughts of CC risk factors are shaped by the culture of the country where they were raised. Palestinian culture encourages having many children as a source of kinship and wealth. Therefore, this might have prevented Palestinian women to consider negative associations with multiparty, such as it being a risk factor of CC. Moreover, more than 70.0% of study participants were married and, considering the high fertility rate in Palestine, which might also have had contributed to shaping such beliefs about multiparity [55]. Education interventions should focus on ‘having many children’ as a risk factor of CC since this is very relevant to the Palestinian society.

### Future directions

The findings of this study reflect the need to promote educational programs to improve women’s knowledge of CC in Palestine. Enriching school curricula with health-related topics and targeting women in the reproductive age should be prioritized. This could drive these women to adjust their behavioral risk factors, hence, decrease their chance of developing CC. In addition, raising young women’s awareness of CC may make them more confident to talk about any possible CC symptom and less embarrassed to seek medical advice or discuss their concerns with healthcare professionals.

### Strengths and limitations

The main strengths of this study included the large sample size and the high response rate. In addition, the stratified approach that may provide a representative view of the target population’s knowledge on different levels of the Palestinian community.

Limitations of this study included the convenience sampling that may limit the generalizability of the findings. However, this may be alleviated by the recruiting a large number of participants while having a high response rate and covering different geographical areas in Palestine. Another limitation could be the exclusion of visitors or patients in the oncology departments and participants with medical backgrounds, possibly decreasing the number of participants with a presumably good level of knowledge. On the other hand, their exclusion was meant to increase the relevancy of this study as a measure of knowledge among the public.

## Conclusion

The overall knowledge of women about CC risk factors was low with only 23.7% of participants demonstrating good knowledge of CC risk factors. Knowing someone with cancer was the only factor associated with an increase in the odds of having good knowledge. Conversely, completing only secondary or diploma degree, being employed, and having a monthly income of ≥ 1450 NIS were all associated with a decrease in the odds of having good knowledge. Introducing topics around sexual and reproductive health, including CC risk factors and symptoms, in school curricula as well as public discourse could be one way of bridging this gap.

## Supplementary Information

**Additional file 1.** Results of all bivariable logistic regression analyses.

## Data Availability

The dataset used and analyzed during the current study is available from the corresponding author upon reasonable request.

## Abbreviations

CC: Cervical cancer
HPV: Human papillomavirus
WBJ: West Bank and Jerusalem
PHCs: Primary healthcare centers
MoH: Ministry of health
CeCAM: Cervical cancer awareness measure
CI: Confidence interval
OR: Odds ratio
STI: Sexually transmitted infection
