# Supplementary material for: Knowledge of cervical cancer risk factors among Palestinian women: a national cross-sectional study
Source: BMC Womens Health. 2021 Nov 2;21:385. doi: 10.1186/s12905-021-01510-2 (PMC8561913; doi:10.1186/s12905-021-01510-2)
Supplement: Supplementary file 1 — Additional file 1. Results of all bivariable logistic regression analyses. [file 12905_2021_1510_MOESM1_ESM.docx]

**Knowledge of Cervical Cancer Risk Factors among Palestinian Women: A National Cross- sectional Study**

Mohamedraed Elshami, MD, MMSc^1,2^*, Mariam Thalji^3^*, Hanan Abukmail, MD^4^*, Ibrahim Al-Slaibi, MD^5^, Mohammed Alser, MD^2^, Afnan Radaydeh^3^, Alaa Alfuqaha^6^, Salma Khader^3^, Lana Khatib^7^, Nour Fannoun, PharmB^8^, Bisan Ahmad^4^, Lina Kassab^2^, Hiba Khrishi^9^, Deniz Elhussaini^10^, Nour Abed^4^, Aya Nammari^3^, Tumodir Abdallah^3^, Zaina Alqudwa^10^, Shahd Idais^3^, Ghaid Tanbouz, DDS^9^, Ma'alem Hajajreh^11^, Hala Abu Selmiyh^4^, Zakia Abo-Hajouj^3^, Haya Hebi^3^, Manar Zamel^7^, Refqa Najeeb Skaik ^10^, Lama Hammoud^9^, Siba Rjoub^3^, Hadeel Ayesh^3^, Toqa Rjoub^3^, Rawan Zakout^4^, Amany Alser^12^, Nasser Abu-El-Noor, PhD^13#^, Bettina Bottcher, MD, PhD^4#^

*Contributed equally as a first co-author.
^#^Contributed equally as a senior co-author.

^1^Harvard Medical School, Boston, Massachusetts, United States.
^2^Ministry of Health, Gaza, Palestine.
^3^Faculty of Medicine, Al-Quds University, Jerusalem, Palestine.
^4^Faculty of Medicine, Islamic University of Gaza, Gaza, Palestine.
^5^Almakassed Hospital, Jerusalem, Palestine.
^6^Faculty of Graduate Studies, An-Najah National University, Nablus, Palestine.
^7^Faculty of Medicine, An-Najah National University, Nablus, Palestine.
^8^Faculty of Pharmacy, Alazhar University of Gaza, Gaza, Palestine.
^9^Faculty of Dentistry and Dental Surgery, Al-Quds University, Jerusalem, Palestine.
^10^Faculty of Medicine, Alazhar University of Gaza, Gaza, Palestine.
^11^Alia Hospital, Hebron, Palestine.
^12^Al-Shiffa hospital, Gaza, Palestine.
^13^Faculty of Nursing, Islamic University of Gaza, Gaza, Palestine.

**Corresponding author**

Mohamedraed Elshami, MD, MMSc
Harvard Medical School, 25 Shattuck Street, Boston, Massachusetts 02115
Phone: 832-245-6055
Email: mohamedraed.elshami@gmail.com

Supplementary table 1: Bivariable logistic regression analyzing the association between recognizing the most identified cervical cancer risk factors and participant characteristics.

| **Characteristic** | **Having a weakened immune system** | | **Infection with a sexually transmitted infection** | | **Infection with HPV** | | **Having a relative with cervical cancer** | | **Long term use of the contraceptive pill** | | **Smoking any cigarettes at all** | |
| --- | --- | --- | --- | --- | --- | --- | --- | --- | --- | --- | --- | --- |
|  | **COR (95% CI)** | **p-value** | **COR (95% CI)** | **p-value** | **COR (95% CI)** | **p-value** | **COR (95% CI)** | **p-value** | **COR (95% CI)** | **p-value** | **COR (95% CI)** | **p-value** |
| **Age group**  18 to 20  21 to 40  41 or older | Ref  1.20 (1.01- 1.44)  1.26 (1.04- 1.53) | Ref  0.041  0.019 | Ref  0.82 (0.67- 0.99)  0.69 (0.56- 0.84) | Ref  0.038  <0.001 | Ref  0.63 (0.53- 0.75)  0.54 (0.45- 0.65) | Ref  <0.001  <0.001 | Ref  1.25 (1.07- 1.46)  1.27 (1.07- 1.50) | Ref  0.005  0.006 | Ref  1.03 (0.88- 1.21)  1.09 (0.92- 1.29) | Ref  0.69  0.34 | Ref  1.26 (1.08- 1.47)  1.34 (1.13- 1.58) | Ref  0.004  0.001 |
| **Educational level**  Illiterate  Primary  Preparatory  Secondary  Diploma  Bachelor  Postgraduate | Ref  1.61 (1.06- 2.44)  2.20 (1.50- 3.24)  2.32 (1.60- 3.37)  1.70 (1.15- 2.51)  2.31 (1.59- 3.34)  3.50 (1.95- 6.29) | Ref  0.026  <0.001  <0.001  0.008  <0.001  <0.001 | Ref  1.08 (0.72- 1.63)  1.79 (1.22- 2.63)  2.07 (1.43- 3.01)  1.63 (1.10- 2.42)  2.16 (1.49- 3.14)  2.79 (1.58- 4.93) | Ref  0.71  0.003  <0.001  0.015  <0.001  <0.001 | Ref  1.06 (0.71- 1.59)  1.23 (0.85- 1.78)  1.43 (0.99- 2.06)  1.48 (1.01- 2.17)  1.75 (1.22- 2.52)  2.10 (1.25- 3.52) | Ref  0.77  0.28  0.051  0.044  0.003  0.005 | Ref  1.14 (0.76- 1.72)  1.14 (0.78- 1.66)  0.96 (0.67- 1.39)  0.74 (0.50- 1.09)  0.91 (0.63- 1.31)  0.98 (0.60- 1.60) | Ref  0.52  0.51  0.84  0.12  0.62  0.93 | Ref  0.92 (0.61- 1.39)  0.95 (0.65- 1.38)  0.92 (0.64- 1.33)  0.82 (0.56- 1.21)  1.09 (0.76- 1.57)  1.54 (0.92- 2.56) | Ref  0.70  0.77  0.67  0.32  0.64  0.10 | Ref  0.94 (0.62- 1.42)  1.06 (0.72- 1.56)  0.82 (0.56- 1.19)  0.70 (0.47- 1.03)  0.67 (0.46- 0.98)  0.78 (0.47- 1.28) | Ref  0.76  0.77  0.29  0.07  0.037  0.33 |
| **Occupation**  Housewife  Employed  Retired  Student | Ref  0.86 (0.75- 0.99)  0.35 (0.22- 0.57)  0.79 (0.67- 0.93) | Ref  0.036  <0.001  0.006 | Ref  0.81 (0.71- 0.93)  0.25 (0.16- 0.41)  1.09 (0.91- 1.30) | Ref  0.002  <0.001  0.34 | Ref  0.94 (0.83- 1.06)  0.51 (0.32- 0.82)  1.81 (1.53- 2.15) | Ref  0.31  0.006  <0.001 | Ref  0.83 (0.73- 0.93)  0.44 (0.27- 0.71)  0.71 (0.61- 0.82) | Ref  0.002  0.001  <0.001 | Ref  1.07 (0.95- 1.21)  0.62 (0.38- 0.99)  1.01 (0.87- 1.17) | Ref  0.24  0.047  0.87 | Ref  0.72 (0.64- 0.81)  0.56 (0.35- 0.90)  0.67 (0.58- 0.78) | Ref  <0.001  0.017  <0.001 |
| **Monthly income**  < 1450 NIS  ≥ 1450 NIS | Ref  0.94 (0.83- 1.06) | Ref  0.30 | Ref  0.92 (0.81- 1.03) | Ref  0.14 | Ref  0.73 (0.65- 0.81) | Ref  <0.001 | Ref  1.13 (1.02- 1.25) | Ref  0.017 | Ref  1.06 (0.96- 1.17) | Ref  0.25 | Ref  0.85 (0.76- 0.94) | Ref  0.001 |
| **Residency**  Gaza Strip  WBJ | Ref  0.74 (0.66- 0.83) | Ref  <0.001 | Ref  0.70 (0.62- 0.78) | Ref  <0.001 | Ref  0.55 (0.50- 0.61) | Ref  <0.001 | Ref  1.16 (1.06- 1.28) | Ref  0.002 | Ref  0.94 (0.85- 1.03) | Ref  0.18 | Ref  0.92 (0.84- 1.02) | Ref  0.10 |
| **Having a chronic disease**  No  Yes | Ref  1.11 (0.96- 1.28) | Ref  0.16 | Ref  0.90 (0.78- 1.03) | Ref  0.12 | Ref  0.82 (0.72- 0.95) | Ref  0.001 | Ref  1.00 (0.89- 1.13) | Ref  0.96 | Ref  1.12 (0.99- 1.26) | Ref  0.06 | Ref  1.11 (0.99- 1.25) | Ref  0.09 |
| **Knowing someone with cancer**  No  Yes | Ref  1.43 (1.28- 1.60) | Ref  <0.001 | Ref  1.19 (1.07- 1.33) | Ref  0.002 | Ref  1.24 (1.12- 1.37) | Ref  <0.001 | Ref  1.32 (1.20- 1.46) | Ref  <0.001 | Ref  1.32 (1.20- 1.46) | Ref  <0.001 | Ref  1.19 (1.08- 1.31) | Ref  <0.001 |
| **Marital status**  Single  Married  Divorced  Widowed | Ref  1.55 (1.37- 1.76)  1.07 (0.74- 1.55)  1.21 (0.86- 1.71) | Ref  <0.001  0.73  0.28 | Ref  1.20 (1.05- 1.36)  0.99 (0.68- 1.45)  0.71 (0.52- 0.99) | Ref  0.006  0.97  0.040 | Ref  0.79 (0.70- 0.89)  0.82 (0.58- 1.17)  0.68 (0.50- 0.93) | Ref  <0.001  0.27  0.016 | Ref  1.42 (1.27- 1.59)  0.92 (0.66- 1.28)  1.19 (0.87- 1.61) | Ref  <0.001  0.62  0.27 | Ref  1.14 (1.02- 1.28)  1.22 (0.87- 1.71)  1.10 (0.81- 1.49) | Ref  0.022  0.25  0.56 | Ref  1.61 (1.44- 1.80)  1.40 (1.01- 1.96)  2.05 (1.49- 2.81) | Ref  <0.001  0.047  <0.001 |
| **Site of data collection**  Public spaces  Hospitals  Primary healthcare centers | Ref  1.03 (0.90- 1.18)  1.50 (1.32- 1.72) | Ref  0.69  <0.001 | Ref  0.71 (0.62- 0.81)  0.94 (0.82- 1.07) | Ref  <0.001  0.36 | Ref  0.69 (0.59- 0.76)  0.58 (0.52- 0.65) | Ref  <0.001  <0.001 | Ref  0.95 (0.84- 1.07)  1.58 (1.41- 1.77) | Ref  0.40  <0.001 | Ref  0.84 (0.75- 0.95)  0.92 (0.82- 1.03) | Ref  0.005  0.14 | Ref  1.21 (1.08- 1.37)  1.74 (1.55- 1.94) | Ref  0.001  <0.001 |

COR= crude odds ratio, CI= confidence interval, WBJ= West Bank and Jerusalem, HPV= Human papillomavirus.

Supplementary table 2: Bivariable logistic regression analyzing the association between recognizing other cervical cancer risk factors and participant characteristics.

| **Characteristic** | **Not going for regular smear (Pap) tests** | | **Having a husband who is not circumcised** | | **Having a husband with many previous partners** | | **Being married at a young age (before age 17)** | | **Having many children (five or more)** | |
| --- | --- | --- | --- | --- | --- | --- | --- | --- | --- | --- |
|  | **COR (95% CI)** | **p-value** | **COR (95% CI)** | **p-value** | **COR (95% CI)** | **p-value** | **COR (95% CI)** | **p-value** | **COR (95% CI)** | **p-value** |
| **Age group**  18 to 20  21 to 40  41 or older | Ref  0.88 (0.75- 1.02)  0.79 (0.67- 0.94) | Ref  0.09  0.007 | Ref  0.80 (0.69- 0.94)  0.68 (0.58- 0.81) | Ref  0.006  <0.001 | Ref  1.00 (0.85- 1.18)  1.19 (0.99- 1.41) | Ref  0.99  0.06 | Ref  0.93 (0.79- 1.10)  1.02 (0.85- 1.22) | Ref  0.43  0.81 | Ref  1.25 (1.03- 1.52)  1.41 (1.14- 1.74) | Ref  0.027  0.001 |
| **Educational level**  Illiterate  Primary  Preparatory  Secondary  Diploma  Bachelor  Postgraduate | Ref  0.95 (0.64- 1.42)  1.13 (0.78- 1.64)  0.97 (0.68- 1.38)  0.84 (0.58- 1.23)  1.02 (0.72- 1.46)  0.90 (0.56- 1.46) | Ref  0.81  0.51  0.86  0.37  0.90  0.68 | Ref  0.82 (0.54- 1.22)  0.82 (0.56- 1.19)  0.87 (0.60- 1.24)  0.71 (0.49- 1.04)  0.96 (0.67- 1.38)  0.95 (0.58- 1.55) | Ref  0.32  0.29  0.44  0.08  0.82  0.83 | Ref  0.87 (0.58- 1.30)  0.68 (0.47- 0.98)  0.53 (0.37- 0.76)  0.50 (0.34- 0.73)  0.50 (0.35- 0.71)  0.63 (0.39- 1.03) | Ref  0.50  0.037  <0.001<0.001  <0.0010.07 | Ref  0.69 (0.45- 1.05)  0.57 (0.39- 0.84)  0.69 (0.48- 1.00)  0.83 (0.56- 1.23)  1.00 (0.69- 1.46)  1.44 (0.88- 2.35) | Ref  0.08  0.005  0.052  0.36  0.98  0.15 | Ref  0.84 (0.53- 1.33)  0.70 (0.46- 1.07)  0.73 (0.49- 1.11)  0.85 (0.55- 1.31)  0.96 (0.64- 1.45)  1.33 (0.78- 2.27) | Ref  0.45  0.10  0.14  0.46  0.85  0.29 |
| **Occupation**  Unemployed/Housewife  Employed  Retired  Student | Ref  0.78 (0.69- 0.87)  0.43 (0.26- 0.72)  0.93 (0.80- 1.07) | Ref  <0.001  0.001  0.29 | Ref  0.89 (0.79- 1.01)  0.58 (0.34- 0.98)  1.37 (1.19- 1.59) | Ref  0.06  0.043  <0.001 | Ref  0.82 (0.73- 0.93)  1.22 (0.75- 1.97)  0.95 (0.82- 1.11) | Ref  0.002  0.42  0.51 | Ref  1.32 (1.17- 1.50)  2.12 (1.32- 3.42)  1.32 (1.13- 1.54) | Ref  <0.0010.002  <0.001 | Ref  1.29 (1.13- 1.48)  1.69 (1.01- 2.82)  0.94 (0.79- 1.13) | Ref  <0.001  0.044  0.51 |
| **Monthly income**  < 1450 NIS  ≥ 1450 NIS | Ref  0.65 (0.59- 0.72) | Ref  <0.001 | Ref  0.87 (0.79- 0.96) | Ref  0.008 | Ref  1.10 (0.99- 1.22) | Ref  0.07 | Ref  1.09 (0.98- 1.21) | Ref  0.13 | Ref  0.99 (0.88- 1.11) | Ref  0.87 |
| **Residency**  Gaza Strip  WBJ | Ref  0.66 (0.59- 0.72) | Ref  <0.001 | Ref  0.85 (0.77- 0.93) | Ref  0.001 | Ref  1.38 (1.25- 1.53) | Ref  <0.001 | Ref  1.14 (1.03- 1.27) | Ref  0.012 | Ref  1.16 (1.03- 1.30) | Ref  0.014 |
| **Having a chronic disease**  No  Yes | Ref  0.90 (0.80- 1.01) | Ref  0.08 | Ref  0.82 (0.73- 0.93) | Ref  0.001 | Ref  1.18 (1.05- 1.33) | Ref  0.006 | Ref  1.09 (0.96- 1.23) | Ref  0.19 | Ref  1.07 (0.93- 1.23) | Ref  0.36 |
| **Knowing someone with cancer**  No  Yes | Ref  1.33 (1.20- 1.46) | Ref  <0.001 | Ref  1.11 (1.01- 1.23) | Ref  0.033 | Ref  1.12 (1.01- 1.24) | Ref  0.028 | Ref  1.04 (0.94- 1.15) | Ref  0.46 | Ref  0.91 (0.81- 1.02) | Ref  0.10 |
| **Marital status**  Single  Married  Divorced  Widowed | Ref  1.07 (0.95- 1.19)  1.19 (0.85- 1.65)  1.01 (0.75- 1.37) | Ref  0.26  0.31  0.95 | Ref  0.97 (0.87- 1.09)  1.08 (0.77- 1.51)  1.04 (0.77- 1.41) | Ref  0.65  0.65  0.80 | Ref  1.18 (1.05- 1.33)  1.21 (0.86- 1.70)  1.23 (0.90- 1.68) | Ref  0.005  0.28  0.19 | Ref  0.78 (0.70- 0.88)  1.12 (0.80- 1.58)  0.93 (0.68- 1.28) | Ref  <0.0010.50  0.66 | Ref  0.88 (0.78- 1.01)  1.58 (1.11- 2.25)  1.18 (0.84- 1.65) | Ref  0.07  0.012  0.35 |
| **Site of data collection**  Public spaces  Hospitals  Primary healthcare centers | Ref  0.94 (0.83- 1.06)  1.52 (1.36- 1.70) | Ref  0.29  <0.001 | Ref  0.83 (0.74- 0.94)  0.84 (0.75- 0.94) | Ref  0.003  0.002 | Ref  1.27 (1.13- 1.43)  0.83 (0.74- 0.93) | Ref  <0.001  0.002 | Ref  0.85 (0.75- 0.96)  0.76 (0.67- 0.85) | Ref  0.010  <0.001 | Ref  0.99 (0.87- 1.15)  0.88 (0.77- 1.00) | Ref  0.93  0.06 |

COR= crude odds ratio, CI= confidence interval, WBJ= West Bank and Jerusalem.
